# Higher emotional awareness is associated with greater domain-general reflective tendencies

**DOI:** 10.1038/s41598-022-07141-3

**Published:** 2022-02-24

**Authors:** Ryan Smith, Michelle Persich, Richard D. Lane, William D. S. Killgore

**Affiliations:** 1 Laureate Institute for Brain Research, 6655 S Yale Ave, Tulsa, OK 74136 USA; 2 Department of Psychiatry, University of Arizona, Tucson, USA; 3 Department of Psychology, University of Arizona, Tucson, USA

**Keywords:** Psychology, Human behaviour

## Abstract

The tendency to reflect on the emotions of self and others is a key aspect of emotional awareness (EA)—a trait widely recognized as relevant to mental health. However, the degree to which EA draws on general reflective cognition vs. specialized socio-emotional mechanisms remains unclear. Based on a synthesis of work in neuroscience and psychology, we recently proposed that EA is best understood as a learned application of domain-general cognitive processes to socio-emotional information. In this paper, we report a study in which we tested this hypothesis in 448 (125 male) individuals who completed measures of EA and both general reflective cognition and socio-emotional performance. As predicted, we observed a significant relationship between EA measures and both general reflectiveness and socio-emotional measures, with the strongest contribution from measures of the general tendency to engage in effortful, reflective cognition. This is consistent with the hypothesis that EA corresponds to the application of general reflective cognitive processes to socio-emotional signals.

## Introduction

Trait differences in emotional awareness (EA) have been the topic of a growing body of empirical work in psychology and psychiatry. Individuals with high EA report granular emotional experiences and perceive similar experiences in others, often promoting more adaptive social and emotional functioning (for a review, see^1^; for related work, see^2^). Current theoretical models posit that the tendency to consciously reflect on the emotions of self and others (e.g., their causes, associated sensations, and how they can be regulated) is a key aspect of EA^3^, as well as of related constructs such as emotional intelligence^4–7^ and alexithymia^8–10^. As measured by the Levels of Emotional Awareness Scale (LEAS;^11,12^), multiple studies suggest that EA is an important determinant of adaptive emotional functioning. High EA has been linked to emotion recognition abilities and openness to experience, among other adaptive skills^11,13–19^. Low EA has also been associated with multiple affective disorders^20–25^. The neurocognitive basis of EA is also an important question within both basic science and clinical research, with a growing number of studies on its developmental basis^16,26,27^ and neural correlates (e.g., for a review, see^28^; for more recent studies, see^23,29–37^).

One important unanswered question pertains to the degree to which the tendency to reflect on emotion in EA depends on domain-general reflective cognitive processes vs. specialized socio-emotional mechanisms. Some models make strong distinctions between emotional and cognitive processes and suggest that the brain contains specialized emotional mechanisms^38,39^; and some neuroscientific studies also suggest the presence of brain regions selectively engaged by social cognition^40–44^. In contrast, other cognitive and neural models suggest less separability between socio-emotional and cognitive process^1,45–49^. In a recent review^50^, we drew on work within evolutionary, developmental, and cognitive neuroscience to argue that EA may have an important dependence on domain-general cognitive processes. Specifically, EA appears to require holding emotional information in mind, integrating it with other available information in perception and memory, and using this information to reflectively plan adaptive courses of action (especially in social situations). While these abilities may be constrained by cognitive capacity (e.g., working memory span, IQ;^32^), it is suggested that trait differences in EA may further depend on the tendency to engage these reflective processes, independent of whether latent cognitive capacity is high or low. In this view, EA involves the application of effortful cognitive processes to emotion-related information (e.g., interoceptive information within oneself, facial, postural, and vocal cues in others, context cues, etc.), which may be facilitated during development by prepared learning and automatic attention biases toward socio-affective signals. The domain-general processes under discussion are “reflective” in the sense that they operate on mental contents in an integrative, slow, and deliberate manner—often reducing the chances of responding maladaptively in emotionally charged situations. However, the degree to which EA depends on domain-general reflective cognitive processes requires further empirical testing.

In this report, we describe a study testing the relationship between EA and measures of both domain-general reflective tendencies as well as emotion-specific skills. We predicted that higher EA would be associated with both the domain-general and emotion-specific measures, as opposed to only the emotion-specific measures (as might be expected if EA were entirely dependent on distinct socio-emotional mechanisms). If this prediction were confirmed, it would support our previously proposed hypothesis regarding how EA emerges through the interaction between affective signals and general reflective cognitive processes.

## Methods

### Participants

A convenience sample of students at the University of Arizona as well as individuals from the surrounding community (mean age = 23.71 years, SD = 5.58; range 18–40), 125 male and 323 female, was recruited from Tucson, AZ. Participants gave informed consent and were paid for their participation. This study was approved by the University of Arizona Institutional Review Board (Protocol #1607696724) and the Human Research and Protection Office of the U.S. Army Medical Research and Development Command (Protocol #A-19136.a and A-19136.b). All methods were performed in accordance with the relevant guidelines and regulations.

### Measures

#### Levels of emotional awareness scale (LEAS).

The 10-tem LEAS^11,13^ asks participants to describe what they believe they and another individual would feel in each of 10 hypothetical scenarios. Freely written responses (typed into a web-based interface; http://eleastest.net/) are scored based solely on the words chosen to describe feelings (scoring is done automatically by computer software). For each scenario, a score of 0 is given to non-emotional words (e.g., confused); a score of 1 is given to words related to bodily sensations (e.g., “exhausted”); a score of 2 is given to words describing emotional actions (e.g., “hitting”) or simple valence distinctions (e.g., “pleasant”, “unpleasant”) that entail approach/avoidance; a score of 3 is given to single emotion concept terms (e.g., “happy,” “afraid”); and a score of 4 is given when at least 2 emotion concept terms are used in a single scenario. For each scenario, the self- and other-related responses are scored separately (i.e., values of 0–4). A “total” score is then given for each scenario, which reflects the higher of the self- and other-related scores. However, if a score of 4 is given for both self and other, a total score of 5 is given for that scenario, as long as the self- and other-related responses are differentiable (for more details see^11^).

#### General reflectiveness measures

We used three related measures to assess general reflective cognition: the cognitive reflection test (CRT-7;^51^), the actively open-minded thinking scale (AOMTS;^52^), and the 2-subscale version of the comprehensive assessment of rational thinking (CART;^53^). The CRT-7 asks seven short questions designed such that there is an immediately intuitive, but incorrect answer, and a correct answer that, while not difficult, requires the individual to devote effortful cognitive resources instead of immediately choosing the intuitively appealing response. Example item:

“If it takes 5 machines 5 minutes to make 5 widgets, how long would it take 100 machines to make 100 widgets?” (intuitive answer: 100 minutes; correct answer: 5 minutes).

It tests the tendency to “stop and think” before immediately trusting one’s intuition.

The AOMTS is a self-report scale which asks individuals to rate 30 statements, from 1 (strongly disagree) to 6 (strongly agree), which describe more or less reflective or “rational” attitudes. Example item:

“I like to gather many different types of evidence before I decide what to do.”

Higher scores indicate more open-minded, reflective attitudes.

The CART assesses vulnerability to various common reasoning biases that arise (in part) from insufficient engagement of reflective capacities. The 2-subscale version we used includes probabilistic/statistical and scientific reasoning problems. Example item:

“Dice game: Even numbers win and odd numbers lose on a die throw. The fair die has six sides, with three even and three odd numbers. Jan has thrown seven odd numbers in a row. What are her chances of throwing an even number on her next throw?” (correct answer: 3/6).

Higher scores indicate a greater tendency to engage effortful cognition and avoid common reasoning biases during problem-solving.

When assessing differences on these reflective cognition measures, it is important to first account for individual differences in general intelligence (IQ). While it is expected that EA will be correlated with IQ (e.g., see^32^), our primary interest is in whether EA is associated with the tendency to automatically engage reflective cognitive processes—independent of differences in latent cognitive capacity. As such, we also asked participants to complete the 2-subscale Wechsler abbreviated scale of intelligence (WASI-II;^54^), a common measure of IQ.

#### Socio-emotional measures

To assess emotion-specific processes, we used the Geneva emotion recognition test (GERT-S;^55^), the brief situational test of emotion management (STEM-B;^56^), and the Mini-K^57^. The GERT-S shows 42 short video clips with audio in which ten actors express 14 different emotions (duration 10 min). After each clip, participants are asked to choose which of the 14 emotions was expressed by the actor. The STEM-B has 18 items, where each item describes an emotional situation. In multiple-choice format (4 choices per item), individuals are asked to select the most effective response for managing both the emotions the person is feeling and the problems presented in each situation. Correct answers are based on norms that were established by a set of solicited subject matter experts. Specifically, they corresponded to the course of action on each item deemed most effective by a group of academics, counselors, psychiatrists, & life coaches with backgrounds in psychology.

The Mini-K is a self-report measure of the amount of cognitive and behavioral resources an individual tends to allocate toward maintaining long-term relationships and future planning more generally—a trait referred to as life history strategy (LHS). It is hypothesized to depend on early learning based on the level of predictability in early childhood environments. For example, an early environment that is safe and includes warm, stable parental/social relationships would be associated with “slow” LHS (high Mini-K scores), where individuals invest heavily in long-term relationships and spend effort planning for the distant future. In contrast, a dangerous early environment (e.g., a low socio-economic status neighborhood with high crime rates) that includes inconsistent or absent parental/social relationships would be associated with a “fast” LHS (low Mini-K scores), where individuals do not tend to maintain long-term relationships and do not focus on distant future outcomes (engaging in more impulsive, risk-seeking behavior). Those with higher Mini-K scores might therefore be expected to show greater LEAS scores due to more experience in stable relationships and/or greater engagement in effortful future-oriented cognition generally, both of which pertain to our hypothesis about the basis of emotional awareness.

As a supplementary measure, we also collected the Toronto Alexithymia Scale (TAS-20;^8^), which is a self-report scale asking participants to provide ratings from 1 (completely disagree) to 5 (completely agree) on 20 items pertaining to difficulties with understanding and expressing emotions. An example item is “I am often confused about what emotion I am feeling”. It includes three subscales reflecting: difficulty identifying feelings, difficulty describing feelings, and externally-oriented thinking. While high alexithymia is conceptually similar to low emotional awareness, a recent meta-analyses found that the TAS-20 and LEAS have a very weak negative relationship with one another (*r* = − 0.12;^58^)—which may reflect differences in measurement approach (i.e., the LEAS does not ask individuals to self-report their emotional awareness), differences in content, and/or that some individuals may not be aware of their limited emotional capacities (see^59^). As our focus is on emotional awareness, we only include the TAS-20 in supplementary analyses to examine whether it is also related to our cognitive and emotional measures in a similar manner to the LEAS.

### Analyses

To assess the relations between EA and each of the cognitive and emotional measures, we first ran JZS Bayes factor analyses with default prior scales in R (BayesFactor package^60,61^) comparing null (intercept only) models to the space of models that included all possible combinations of main effects of age, sex, IQ, and the predictor of interest on LEAS total scores. Because there were significant or marginal differences between males and females for all cognitive and emotional measures (see Table 1), Bayes factors (BFs) were also calculated in relation to models with possible interactions between sex and these other measures. A BF represents the ratio of the probability of the data under one model vs. another, indicting the relative evidence for different models. If ${H}_{0}$ indicates the null hypothesis, ${H}_{1}$ indicates the alternative hypothesis, and $d$ indicates the data, then:

**Table 1:** Summary statistics (mean and SD) for study measures by sex.

	Usable data (N)*	Total	Female	Male	Effect of Sex**
Age	Female: 323 Male: 125 Total: 448	23.7 (5.58)	23.39 (5.49)	24.5 (5.74)	*t*(446) = − 1.9, *p* = 0.06, *d* = − 0.18; BF = 0.65
LEAS	Female: 292 Male: 113 Total: 405	38.54 (4.97)	39.44 (4.73)	36.19 (4.83)	*t*(403) = 6.16, ***p* < 0.001**, *d* = 0.61; BF > 100
IQ	Female: 320 Male: 125 Total: 445	108.57 (11.52)	107.97 (11.25)	110.11 (12.1)	*t*(443) = − 1.77, *p* = 0.08, *d* = − 0.17; BF = 0.52
CART	Female: 319 Male: 124 Total: 443	10.52 (2.47)	10.06 (2.26)	11.7 (2.58)	*t*(441) = − 6.57, ***p* < 0.001**, *d* = − 0.63; BF > 100
CRT-7	Female: 311 Male: 119 Total: 430	2.67 (2.09)	2.31 (1.93)	3.62 (2.19)	*t*(428) = − 6.1**, *p* < 0.001**, *d* = − 0.59; BF > 100
AOMTS	Female: 321 Male: 125 Total: 446	134.65 (13.41)	133.35 (13.05)	137.98 (13.78)	*t*(444) = − 3.31, ***p* = 0.001**, *d* = − 0.31; BF = 21.61
STEM-B	Female: 312 Male: 121 Total: 433	0.63 (0.08)	0.64 (0.08)	0.62 (0.09)	*t*(431) = 1.7, *p* = 0.09, *d* = 0.16; BF = 0.47
GERT-S	Female: 299 Male: 113 Total: 412	26.46 (4.12)	26.71 (4.11)	25.8 (4.1)	*t*(410) = 2.01, ***p* = 0.04**, *d* = 0.2; BF = 0.84
MINI-K	Female: 317 Male: 123 Total: 440	24.09 (12.46)	25.3 (12.5)	20.97 (11.85)	*t*(438) = 3.31, ***p* = 0.001**, *d* = 0.32; BF = 21.46
TAS-20	Female: 320 Male: 125 Total: 445	42.65 (10.91)	42.95 (11.29)	41.88 (9.85)	*t*(443) = 0.93, *p* = 0.35, *d* = 0.09; BF = 0.18

*Not all data were available from all participants. Outlier values greater than three standard deviations from the mean were also removed. We therefore report final available Ns for each measure.

**Significant sex differences at p < .05 are bolded for clarity.

$$BF= \frac{p(d|{H}_{1})}{p(d|{H}_{0})}$$

Higher BF values indicate greater evidence for the alternative hypotheses; e.g., BF = 3 indicates three times as much evidence for the alternative hypothesis than for the null hypothesis (i.e., the data are three times as probable under the alternative hypothesis). We adopt the guidelines described in Lee and Wagenmakers^62^ for interpreting the strength of evidence provided by different BF values: BF = 1–3, poor/anecdotal evidence; 3–10, moderate evidence; 10–30, strong evidence, 30–100, very strong evidence, > 100, extremely strong evidence. Here we focus on Bayesian analyses because they have important statistical advantages. Namely, BFs provide a straightforward means of model comparison, their values do not require correction for multiple comparisons, and they are able to assess evidence for the null model as well as alternative models^63–65^.

After identifying the model with the most evidence, we conducted post-hoc Pearson correlations to assess the strength of relationships between each predictor variable and LEAS total scores. After confirming the hypothesized relationships, we then performed secondary correlational and hierarchical clustering analyses to further characterize relationships between our domain-general and socio-emotional measures (using the agglomerative complete linkage method within the ‘hclust’ function and ‘corrplot’ package in R; https://cran.r-project.org/web/packages/fastcluster/fastcluster.pdf). These secondary analyses were performed to best interpret the specific nature of the relationships supported by our initial results. Although not the focus of our primary analyses, we also explored whether the TAS-20 might show a similar pattern of relationships to the LEAS. For directional consistency when performing clustering, TAS-20 scores were inverted such that higher scores indicated lower alexithymia (i.e., higher self-reported emotional awareness).

### Informed consent

Informed consent was obtained from all individual participants included in the study.

## Results

The descriptive statistics for all variables are shown in Table 1, which also includes comparisons between males and females. As seen there, LEAS, GERT-S, and MINI-K scores were greater in females than in males, while CRT-7, AOMTS, and CART scores were greater in males than in females.

### General reflectiveness measures

#### CART

**Figure 1:**
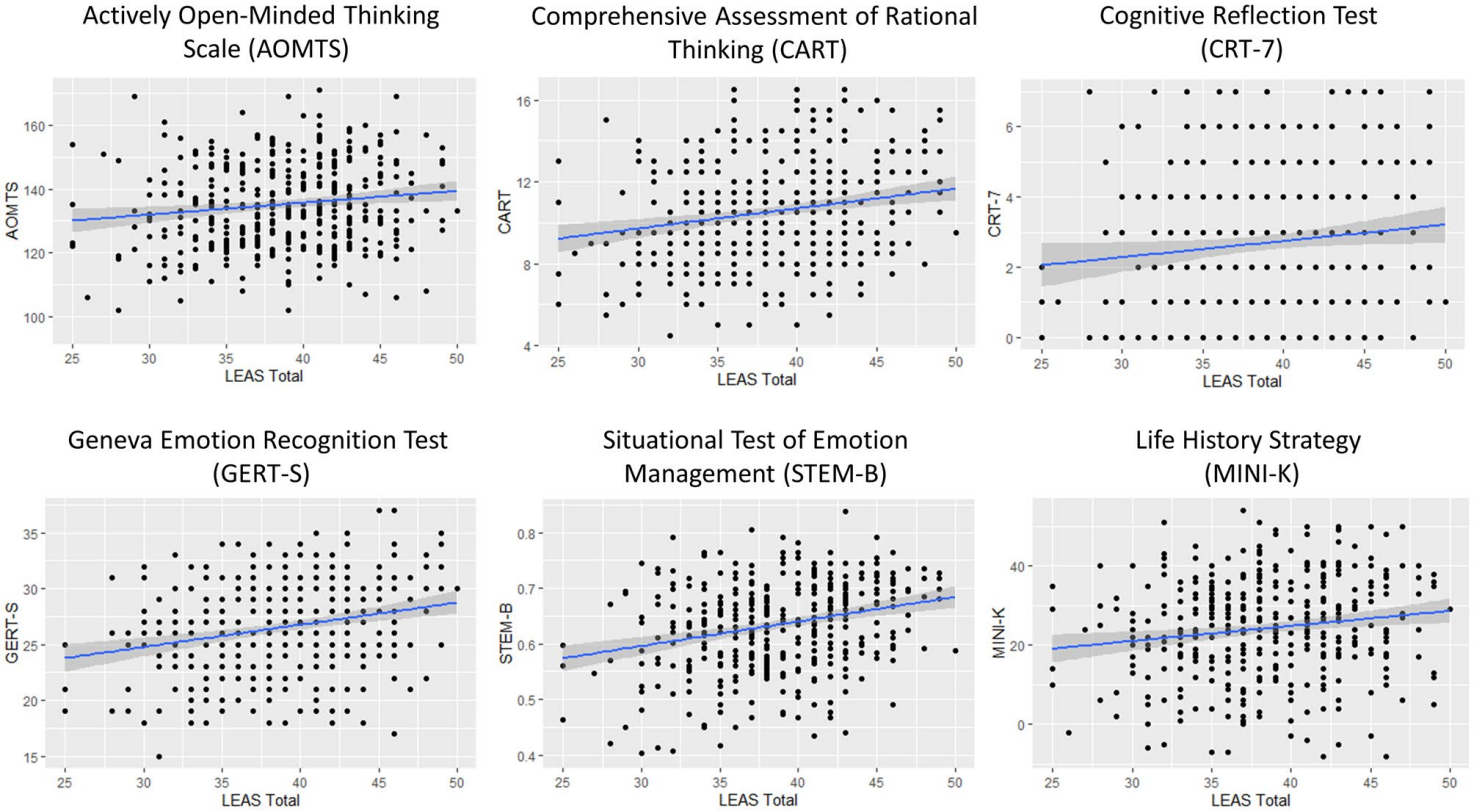
Scatterplots illustrating the relationship between LEAS total scores and three general reflective cognition measures (AOMTS, CART, CRT-7), as well as three socio-emotional cognition measures (GERT-S, STEM-B, Mini-K).

Bayes factor analyses comparing models including all possible combinations of age, sex, IQ, and CART total scores (and interactions between sex and both IQ and CART) revealed the most evidence for a model with sex, IQ, and CART total scores as predictors of LEAS total scores (Bayes factor [BF] > 100 relative to an intercept-only model; extremely strong evidence). This model had moderate evidence (BF = 3.50) relative to the 2nd-best model, which also included an interaction between sex and IQ. It also had very strong evidence (BF = 78.08) relative to a model with only sex and IQ. Posterior regression coefficients for the best model were: sex (female): b = 1.96, 95% credible interval [CI] = [1.44 2.48]; IQ: b = 0.08, CI = [0.03 0.12]; CART: b = 0.41, CI = [0.19 0.63]. A post-hoc Pearson correlation analysis showed the hypothesized positive relationship between CART total scores and LEAS total scores (*r* = 0.20, *p* < 0.001, BF > 100; see Fig. 1 for a scatterplot depiction). Due to the sex differences observed in both variables, we also examined these correlations separately in each sex (males: *r* = 0.39, *p* < 0.001, BF > 100; females: *r* = 0.28, *p* < 0.001, BF > 100). These correlations were not significantly different between males and females (as assessed using the r.test function within the psych package in R; https://CRAN.R-project.org/package=psych).

#### AOMTS

Bayes factor analyses comparing models including all possible combinations of age, sex, IQ, and AOMTS total scores (and interactions between sex and both IQ and AOMTS) revealed the most evidence for a model with sex, IQ, and AOMTS total scores (BF > 100 relative to an intercept-only model; extremely strong evidence). However, this model had poor evidence (BF = 1.06) relative to the 2nd-best model, which removed AOMTS. Posterior regression coefficients for the best model were: sex (female): b = 1.74, 95% credible interval [CI] = [1.24 2.25]; IQ: b = 0.11, CI = [0.07 0.15]; AOMTS: b = 0.04, CI = [0.002 0.07]. A post-hoc Pearson correlation analysis showed the hypothesized positive relationship between AOMTS total scores and LEAS total scores (*r* = 0.14, *p* = 0.001, BF = 6.6; see Fig. 1). Due to the sex differences observed in both variables, we also examined these correlations separately in each sex (males: *r* = 0.16, *p* = 0.09, BF = 0.84; females: *r* = 0.20, *p* < 0.001, BF = 46.2). These correlations were not significantly different between males and females.

#### CRT-7

Bayes factor analyses comparing models including all possible combinations of age, sex, IQ, and CRT-7 total scores (and interactions between sex and both IQ and CRT-7) revealed the most evidence for a model with sex and IQ as predictors of LEAS total scores (BF > 100 relative to an intercept-only model; extremely strong evidence). This model had moderate evidence (BF = 5.04) relative to a model that added CRT-7. Posterior regression coefficients for the model including CRT-7 were: sex (female): b = 1.66, 95% credible interval [CI] = [1.31 2.16]; IQ: b = 0.11, CI = [0.07 0.16]; CRT-7: b = 0.11, CI = [− 0.14 0.38]. A post-hoc Pearson correlation analysis showed a positive relationship between CRT-7 scores and LEAS total scores (*r* = 0.11, *p* = 0.03, BF = 1.11; see Fig. 1). Due to the sex differences observed in both variables, we also examined these correlations separately in each sex (males: *r* = 0.21, *p* = 0.03, BF = 1.91; females: *r* = 0.19, *p* < 0.001, BF = 27.00). Thus, hypothesized relationships were present, but may be accounted for by differences in IQ. The correlations were not significantly different between males and females.

### Socio-emotional measures

#### GERT-S

Bayes factor analyses comparing models including all possible combinations of age, sex, IQ, and GERT-S scores (and interactions between sex and both IQ and GERT-S) revealed the most evidence for a model with sex, IQ, and GERT-S scores as predictors of LEAS total scores (BF > 100 relative to an intercept-only model; extremely strong evidence). This model had moderate evidence (BF = 3.26) relative to the 2nd-best model, which removed GERT-S. Posterior regression coefficients for the best model were: sex (female): b = 1.54, 95% credible interval [CI] = [1.02 2.04]; IQ: b = 0.11, CI = [0.06 0.15]; GERT-S: b = 0.15, CI = [0.04 0.27]. A post-hoc Pearson correlation analysis showed the hypothesized positive relationship between GERT-S scores and LEAS total scores (*r* = 0.24, *p* < 0.001, BF > 100; see Fig. 1). Due to the sex differences observed in both variables, we also examined these correlations separately in each sex (males: *r* = 0.21, *p* = 0.04, BF = 1.75; females: *r* = 0.23, *p* < 0.001, BF > 100). The correlations were not significantly different between males and females.

#### STEM-B

Bayes factor analyses comparing models including all possible combinations of age, sex, IQ, and STEM-B scores (and interactions between sex and both IQ and STEM-B) revealed the most evidence for a model with sex, IQ, and STEM-B total scores as predictors of LEAS total scores (BF > 100 relative to an intercept-only model; extremely strong evidence). This model had moderate evidence (BF = 3.75) relative to the 2nd-best model, which also included an interaction between IQ and STEM-B, and strong evidence (BF = 43.19) relative to a model with only sex and IQ (i.e., removing STEM-B). Posterior regression coefficients for the best model were: sex (female): b = 1.53, 95% credible interval [CI] = [1.05 2.01]; IQ: b = 0.10, CI = [0.06 0.14]; STEM-B: b = 9.93, CI = [4.43 15.66]. A post-hoc Pearson correlation analysis showed the hypothesized positive relationship between STEM-B scores and LEAS total scores (*r* = 0.27, *p* < 0.001, BF > 100; see Fig. 1). Due to the sex differences observed in both variables, we also examined these correlations separately in each sex (males: *r* = 0.19, *p* = 0.05, BF = 1.49; females: *r* = 0.29, *p* < 0.001, BF > 100). The correlations were not significantly different between males and females.

#### Mini-K

Bayes factor analyses comparing models including all possible combinations of age, sex, IQ, and Mini-K scores (and interactions between sex and both IQ and Mini-K) revealed the most evidence for a model with sex, IQ, and Mini-K scores as predictors of LEAS total scores (BF > 100 relative to an intercept-only model; extremely strong evidence). However, this model had poor evidence (BF = 2.04) relative to the 2^nd^-best model, which removed the Mini-K. Posterior regression coefficients for the best model were: sex (female): b = 1.60, 95% credible interval [CI] = [1.09 2.11]; IQ: b = 0.12, CI = [0.08 0.16]; Mini-K: b = 0.04, CI = [0.006 0.08]. A post-hoc Pearson correlation analysis showed the hypothesized positive relationship between Mini-K scores and LEAS total scores (*r* = 0.15, *p* < 0.001; see Fig. 1). Due to the sex differences observed in both variables, we also examined these correlations separately in each sex, showing a numerically stronger relationship in females (males: *r* = 0.06, *p* = 0.56, BF = 0.26; females: *r* = 0.13, *p* = 0.003, BF = 1.49). The correlations were not significantly different between males and females.

### Joint model

Having found evidence in separate models for the contribution of both general reflectiveness and socio-emotional measures, we then included all contributing measures in a single model to assess the relative importance of each measure. Here we only included main effects found in the winning models above, due to the very large set of models generated if including all possible main effects and interactions. This revealed the most evidence for a model with sex, CART, GERT-S, and STEM-B scores as predictors of LEAS total scores (BF > 100 relative to an intercept-only model; extremely strong evidence). However, this model had poor evidence relative to the 2nd- to 12th-best models (BFs = 1.37 to 2.69). All of these models included sex, CART, and STEM-B, but variably added IQ, CRT-7, and/or Mini-K, or removed GERT-S. To assess the importance of each predictor in the winning model, we compared it to models in which each term was removed. This revealed that all variables but GERT-S made an important contribution. In descending order, the winning model had BFs > 100 compared to models where sex or CART were removed, BF = 85.89 when STEM-B was removed, and BF = 1.4 when GERT-S was removed. Posterior regression coefficients for the winning model were: sex (female): b = 1.74, 95% credible interval [CI] = [1.21 2.27]; CART: b = 0.46, CI = [0.27 0.67]; GERT-S: b = 0.12, CI = [0.01 0.23] ]; STEM-B: b = 10.53, CI = [4.67 16.46].

### Post-hoc correlations and associations with alexithymia

After completing our primary analyses, we ran a series of post-hoc correlations to provide a more complete characterization of relationships between all variables. Although performed primarily to characterize previously identified results, for the interested reader we note uncorrected p-value thresholds and BFs for associated Bayesian correlation analyses. As a further exploratory analysis, we also examined correlations between these measures and TAS-20 scores to assess whether self-reported difficulties associated with emotional awareness (i.e., alexithymia) might show a similar pattern as found for the LEAS (which is not based on self-perceived traits). Although post-hoc, we note that a sensitivity analysis, performed using G*Power3^66^, indicated that our sample of 447 participants was able to detect a small effect size of ρ = 0.13 for correlations between our variables of interest, assuming a power of 0.80 and α = 0.05 (two-sided).

**Figure 2:**
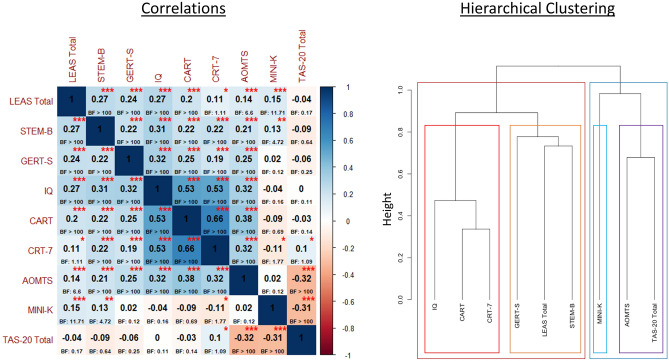
Left: Post hoc Pearson correlations between all variables. Although carried out post-hoc to further characterize results of prior planned analyses, for the interested reader we note their uncorrected significance levels (*p < .05, **p < .01, ***p < .001) and provide Bayes factors (BFs) from JZS Bayesian correlation analyses with default prior scales in R (see main text) indicating the level of evidence for each relationship. Right: Results of hierarchical clustering analyses. The LEAS, IQ, two performance-based general reflectiveness measures (CART, CRT-7), and two performance-based socio-emotional variables (STEM-B, GERT-S) clustered together with significant positive correlations; finer-grained clustering grouped the LEAS with STEM-B and GERT-S. The Mini-K clustered with a self-report reflectiveness measure (AOMTS) and a self-report measure of alexithymia (TAS-20) with negative correlations; finer-grained clustering separated the Mini-K from these other two variables. Note that clustering analyses were performed after making TAS-20 scores negative so that higher scores indicated higher levels of emotional awareness.

The left panel of Fig. 2 shows the resulting post-hoc correlation matrices. The right panel of Fig. 2 also shows results of a hierarchical clustering analysis on this correlation matrix. Notably, the STEM-B and GERT-S were both positively correlated with the general reflectiveness measures, and all performance-based measures clustered (all positive correlations) separately from self-report measures—where AOMTS and Mini-K were negatively correlated with TAS-20 (i.e., higher alexithymia was associated with less self-reported reflectiveness and faster LHS). TAS-20 was not correlated with LEAS.

For further interpretation, Supplementary Fig. 1 also shows relationships between all measures and the subscales for the LEAS, CART, and TAS-20 (see Supplementary Table S1 for descriptive statistics). As seen there, the pattern of relationships with LEAS subscale scores and CART subscale scores was similar to that observed for total scores. Notably, the TAS-20 externally-oriented thinking subscale showed negative associations with the LEAS and multiple cognitive and socio-emotional measures.

## Discussion

Based on a theoretical model we recently proposed^50^, we tested the prediction that higher EA would be associated with the general tendency to engage in effortful/reflective cognition. We also tested relationships with other socio-emotional measures more traditionally expected to be associated with EA. In support of our predictions, positive associations were observed between EA (LEAS) and all measures of both reflective cognition and socio-emotional skills—with Bayesian analyses finding the strongest evidence for models that included sex, IQ, one measure of reflective cognition (CART) and two socio-emotional measures (GERT-S, STEM-B). When comparing models including all combinations of measures, the evidence suggested that, after sex, the CART was of highest importance, followed by a strong additional contribution of STEM-B. These results are consistent with our previous proposal^50^ that EA draws on domain-general reflective cognitive capacities to a significant degree, as opposed to depending primarily on specialized socio-emotional mechanisms.

As observed previously, EA was positively correlated with IQ (e.g.,^32^), Here it is important to emphasize, however, that the effect of the CART was found after accounting for differences in IQ. This means that the relationship between reflectiveness measures and EA is not explained by differences in general cognitive ability. Instead, it corresponds to the general habit or tendency to *engage* those cognitive capacities when making decisions. Interestingly, post-hoc correlations showed that the other socio-emotional measures were also associated with the general cognitive measures, suggesting an important contribution of domain-general cognition to socio-emotional processes more broadly.

Post-hoc analyses separating self-focused and other-focused EA did not identify unique patterns from total EA scores. Despite the sex differences in EA (higher in females) and in general effortful cognition (higher in males)—both of which replicate previous findings^19,53,67,68^—relationships between these measures were largely similar in both males and females. This suggests these relationships may be quite general, and not driven by overlap in a circumscribed set of narrower sub-processes. One exception was the Mini-K, where results suggested the relationship with EA may be driven more by females. This is consistent with a previous study finding relationships between EA and another more comprehensive measure of life history strategy (the K-SF-42;^69^).

Finally, we performed supplementary analyses examining whether a self-report measure of alexithymia (TAS-20), which is conceptually similar to the construct of emotional awareness, would show a similar pattern of relationships to that observed for the LEAS. Consistent with previous literature^58^, LEAS and TAS-20 scores showed very weak negative associations; and the TAS-20 showed a unique pattern of relationships with other measures—primarily a negative relationship with self-report measures of reflective cognition (AOMTS) and life history strategy (Mini-K). Overall these findings are theoretically consistent with our prediction about the relationships between emotional awareness and reflective cognition, but also highlight the way that experimental operationalizations of (self-reported) alexithymia and (performance-based) emotional awareness can produce distinct patterns of results.

It is important to consider the strengths and limitations of this study. Major strengths include a sufficiently powered sample size to detect small effect sizes, the use of multiple convergent measures, and incorporation of Bayesian analyses facilitating model selection. One weakness is that there were fewer men than women, affording less confidence in observed relationships when examining men separately. Some measures could have also favored men (e.g., mathematical reasoning on the CART/CRT-7) or women (e.g., maintaining long-term relationships on the Mini-K) due to influences of socialization and sex-specific societal values. Future research should therefore replicate our results using convergent measures with fewer potential influences of this kind (e.g., using non-mathematical reasoning measures). Another weakness is that the study design only affords correlational analyses. It can therefore not provide a strong test of hypotheses about the way in which general reflective cognition interacts with specialized socio-emotional signal processing mechanisms to allow for high EA; nor can it test hypotheses about how these processes interact to facilitate acquisition of EA during development. These interactions are supported by prior neuroimaging studies linking EA with interactions between domain-general cognitive control networks while individuals attend to and hold emotions in mind (and in resting conditions)^28–32,34^—but future research will be needed to address these questions further.

Aside from a self-report measure of alexithymia, another limitation is that we did not incorporate other measures related to emotional awareness, such as measures of interoceptive awareness^70–75^, emotional granularity^2^, or emotional intelligence^5,76^. Granular emotion terms receive higher scores on the LEAS, so it is plausible to hypothesize that other granularity measures could show a similar pattern of results to those found here. The construct of emotional intelligence incorporates a number of traits/skills beyond EA (e.g., emotion recognition and regulation)—and there are several different self-report and performance-based measures (and competing models) of emotional intelligence (for a review of different models, and their potential neural basis, see^4^). Thus, while it would also be useful for future research to examine the way emotional intelligence relates to reflective cognition, this would not directly address the theoretical hypothesis motivating the present study. A final limitation is that this is the first study to look at the relationships between measures of EA and reflective cognition, and our participants had a restricted age range and were sampled from a single geographic region. Thus, replication and extension to a broader range of demographics will be necessary to confirm the existence and generalizability of our findings.

In summary, the combination of findings in this study are consistent with the idea that acquiring high EA—either during development or later in life ^16,77–82^—depends on developing general tendencies to engage in effortful, reflective cognitive processes. They also suggest a significant overlap between domain-general cognition and socio-emotional capacities more broadly. This adds to a growing body of work, both in neuroscience and psychology^45,46,48,83,84^, suggesting a strong overlap in the mechanisms underlying cognitive and emotional processes.

## Supplementary Information

Supplementary Information.
