# Supplementary material for: Higher emotional awareness is associated with greater domain-general reflective tendencies
Source: Sci Rep. 2022 Feb 24;12:3123. doi: 10.1038/s41598-022-07141-3 (PMC8873306; doi:10.1038/s41598-022-07141-3)
Supplement: Supplementary file 1 — Supplementary Information. [file 41598_2022_7141_MOESM1_ESM.docx]

**Supplemental Materials:**

**Higher emotional awareness is associated with greater domain-general reflective tendencies**

Ryan Smith^1^, Michelle Persich^2^, Richard D. Lane^2,3^, William D.S. Killgore^2,3^

^1^ Laureate Institute for Brain Research

^2^ Department of Psychiatry, University of Arizona

^3^ Department of Psychology, University of Arizona

**Supplementary Results**

**Table S1. Summary statistics (mean and SD) for subscales**

| **Measures** | **Usable Data (N)** |  | **Total** | **Female** | **Male** | **Effect of Sex** |
| --- | --- | --- | --- | --- | --- | --- |
|  |  |  | 448 | 323 | 125 |  |
| LEAS Self | Female: 292 Male: 113 Total: 405 |  | 32.57 (4.52) | 33.45 (4.06) | 30.3 (4.87) | *t(*403) = 6.62, *p <* 0.001, *d =* 0.66; BF > 100 |
| LEAS Other | Female: 292 Male: 113 Total: 405 |  | 31.27 (4.32) | 31.83 (4.08) | 29.82 (4.6) | *t(*403) = 4.28, *p <* 0.001, *d =* 0.43; BF > 100 |
| CART Prob. Stat. Reasoning | Female: 320 Male: 125 Total: 445 |  | 9.06 (2.63) | 8.58 (2.43) | 10.28 (2.72) | *t(*443) = -6.41, *p <* 0.001, *d =* -0.61; BF > 100 |
| CART Sci. Reasoning | Female: 319 Male: 125 Total: 444 |  | 11.95 (3.05) | 11.54 (2.87) | 13 (3.25) | *t(*442) = -4.64, *p <* 0.001, *d =* -0.44; BF > 100 |
| TAS-20 DIF | Female: 320 Male: 125 Total: 445 |  | 13.42 (5.31) | 13.7 (5.4) | 12.72 (5.02) | *t(*443) = 1.75, *p =* 0.08, *d =* 0.17; BF = .51 |
| TAS-20 DDF | Female: 320 Male: 125 Total: 445 |  | 11.68 (4.68) | 11.74 (4.92) | 11.54 (4) | *t(*443) = 0.41, *p =* 0.68, *d =* 0.04; BF = .13 |
| TAS-20 EOT | Female: 320 Male: 125 Total: 445 |  | 17.55 (3.77) | 17.52 (3.79) | 17.62 (3.71) | *t(*443) = -0.27, *p =* 0.79, *d =* -0.03; BF = .12 |


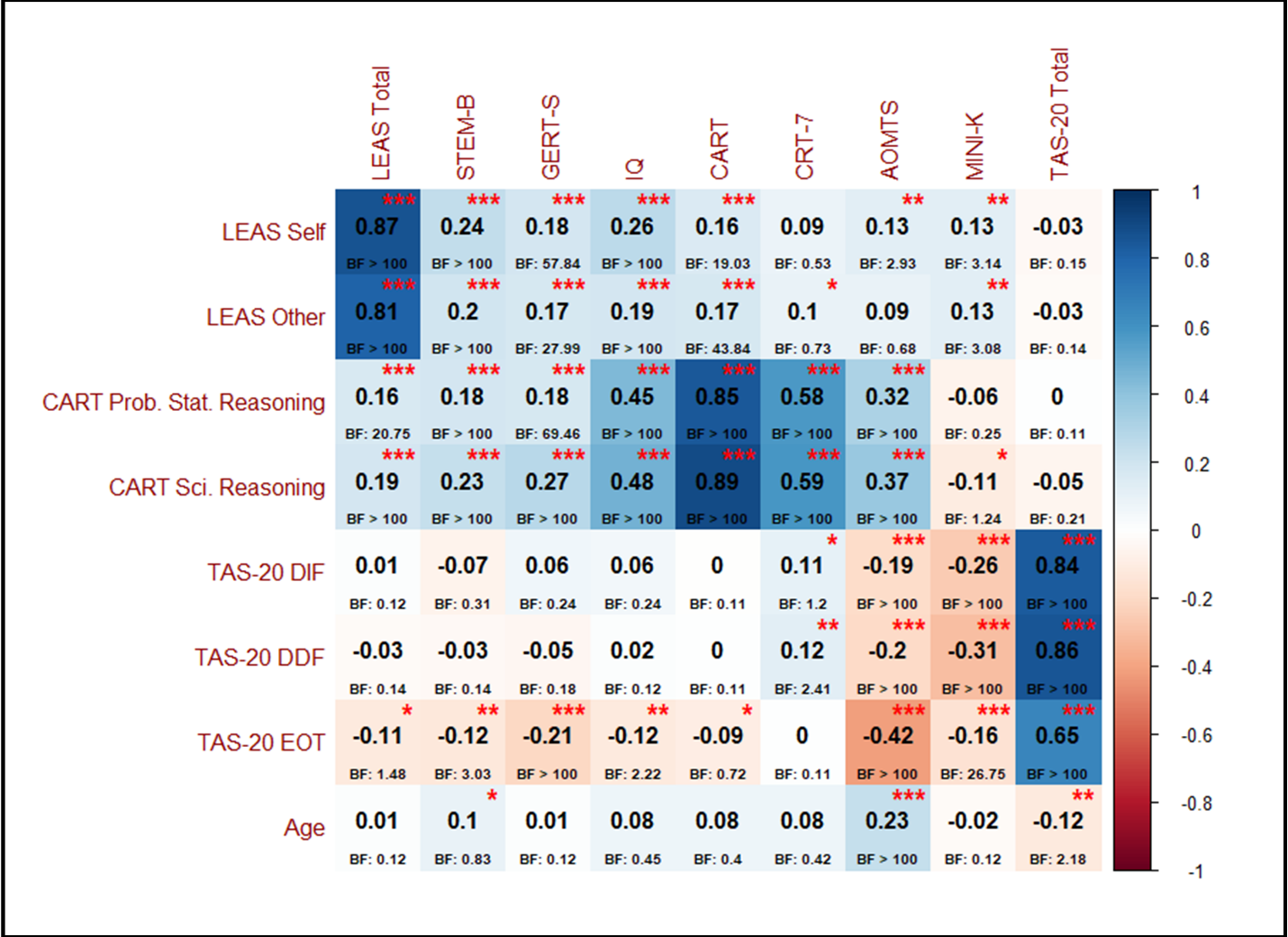
**Figure S1.** Post hoc Pearson correlations between all the LEAS, CART, and TAS-20 subscales and all other measures. Although carried out post-hoc to further characterize results of prior planned analyses, for the interested reader we note their uncorrected significance levels (* = p < .05, ** p < .01, *** p < .001) and provide Bayes factors (BFs) from JZS Bayesian correlation analyses with default prior scales in R (see main text) indicating the level of evidence for each relationship. Relationships were largely similar as with the total scores for these scales. Notably, however, the TAS-20 externally oriented thinking (EOT) subscale showed significant negative correlations with LEAS and multiple cognitive and socio-emotional measures. DIF = difficulty identifying feelings; DDF = difficulty describing feelings.
